# Spontaneous Spinal Epidural Hematoma in a 12-Year-Old Child

**DOI:** 10.7759/cureus.16728

**Published:** 2021-07-29

**Authors:** Abdullah S Alkhuraiji, Osama A Alrehaili, Ahmad A Al Boukai

**Affiliations:** 1 Department of Orthopaedic Surgery, King Saud University Medical City, Riyadh, SAU; 2 Department of Radiology, King Saud University Medical City, Riyadh, SAU

**Keywords:** cervical spine, epidural hematoma, children, spinal cord injury, trauma

## Abstract

Spontaneous spinal epidural hematoma (SSEH) is uncommon, with an estimated incidence of one per million per year in the general population. Since SSEH was first described, only 29 cases have been reported in children. This condition is difficult to diagnose and needs immediate surgical intervention for hematoma evacuation and cord decompression to obtain optimal functional and neurological outcomes. The presentation in children might be atypical. We present a case that was managed surgically and yielded full recovery.

## Introduction

Spontaneous spinal epidural hematoma (SSEH) is a disorder characterized by accumulation of blood in the epidural space, which puts pressure and compression on the spinal cord. The first case was reported and described in 1869 by Jackson [1]. The incidence has been estimated at one per million per year in the general population [2]. Only 29 cases have been reported in the pediatric population and the majority of the reported cases spontaneously occurred [3-5]. These reports showed the importance of early detection and emergency hematoma evacuation to achieve practical outcomes [3,6,7]. SSEH symptoms include an acute onset of severe neck pain which might be associated with mid back pain followed by symptoms of rapidly evolving nerve root or spinal cord compression. Clinically, people affected had a wide range of presentation from radiculopathy symptoms to quadriplegia depending on the haematoma size and site of cord compression. The presentation in children may vary or be atypical which is mainly affected by the age of the patient [8-11]. SSEH's true etiology is unknown but several associations between SSEH and conditions such as coagulopathy, arteriovenous malformation and use of anticoagulants have been reported [12,13]. In this case, we are presenting a late presentation case of spontaneous spinal epidural hematoma treated with surgical intervention and resulting in complete recovery.

## Case presentation

A 12-year-old female presented to our emergency with history of neck pain for seven days associated with gradual left side upper and lower limb weakness and numbness that affected her ability to walk. She denied any history of trauma, fever, loss of weight, urinary and bowel symptoms. 

At the start of her symptoms, she was managed in a small community hospital as a case of meningitis. Brain and cervical CT scans were unremarkable. An empirical antibiotic was commenced with no improvement. As weakness progressed, patient was referred to the emergency department in our institute.

On presentation, the child was alert. She had an oral temperature of 37.3°C, 22/minute respiratory rate, 85/minute pulse rate, and 114/56 mmHg blood pressure and 97% oxygen saturation on room air. Her motor examination revealed intact motor power for right upper extremity (5/5), weakness of right lower extremity (3/5), and weakness of upper and lower left extremities (1/5). No sensory deficits were present. The patient’s deep tendon reflexes were only appreciated in the right lower limb. Her rectal tone and peri-anal sensation were intact. No other upper motor neuron lesion signs were detected. 

**Figure 1:**
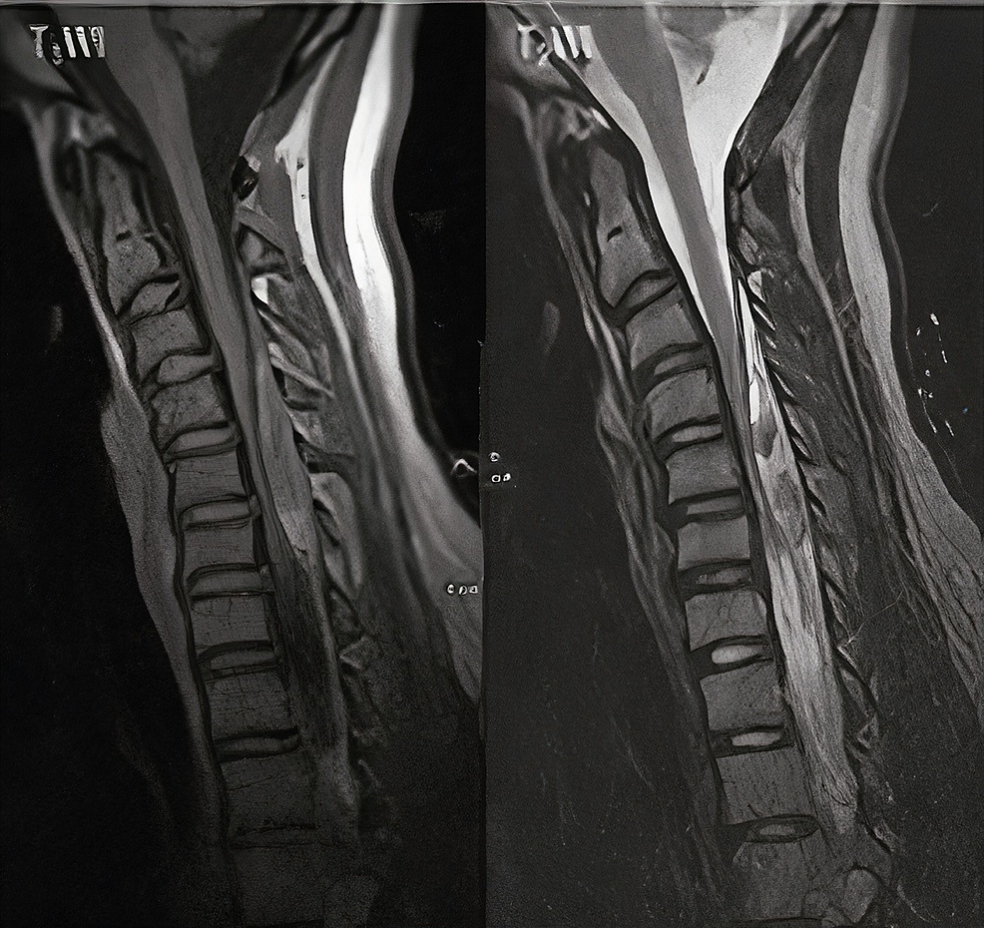
Sagittal MR images in T1WI & T2WI A heterogenous fusiform long left posterolateral epidural lesion that appears generally isointense on T1W, and heterogeneously high on T2W images with focal areas of altered signal within. No remarkable contrast enhancement may could be seen. Findings are in keeping with epidural hematoma.

**Figure 2:**
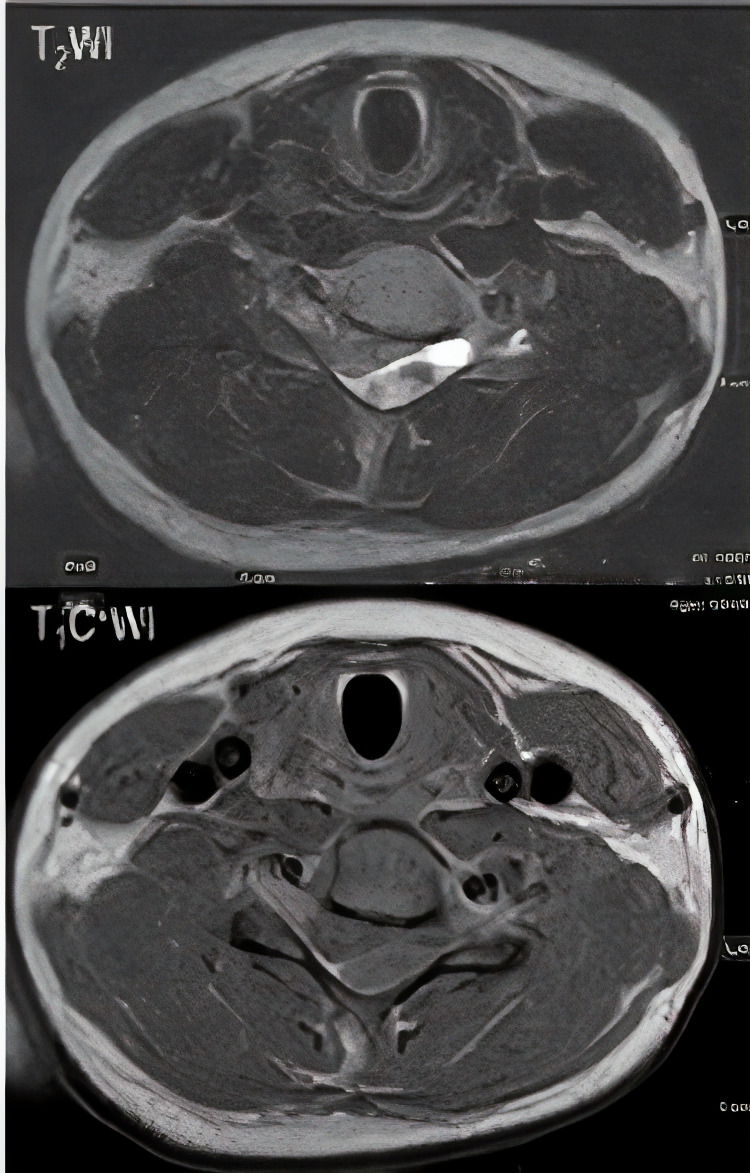
Axial MR images at same level in T2WI & T1 post contrast enhancement A heterogenous fusiform left posterolateral epidural lesion that appears heterogeneously high on T2W images with no remarkable contrast enhancement in the post gadolinium T1 study. Findings are in keeping with epidural hematoma.

Lab tests revealed white blood cell (WBC) = 8900/μl, platelet count = 457000/μl, hemoglobin = 12.8 mg/dl, sodium = 136 mEq/L, potassium = 4.4 mEq/L, erythrocyte sedimentation rate (ESR) = 11 millimeter/hour, prothrombin time (PT) = 14 seconds, partial thromboplastin time (PTT) = 33.2 seconds, and international normalized ratio (INR) = 1.01. An urgent MRI showed posterolateral space-occupying epidural lesion which appears isointense on T1-weighted and hyper-intense on T2-weighted images without enhancement of contrast in the post gadolinium T1 study causing a spinal cord compression (Figures 1, 2).

The patient was hospitalized in our institute, an emergent posterior cervical decompression and evacuation of hematoma from C4-6 along with instrumented fusion from C3-6 with bone grafting. Our aim was to start with partial laminectomy but unfortunately it was not feasible to evacuate the hematoma due to blood clots and this finding lead to do full laminectomy from C4-C6. There was no obvious pus or vascular malformation identified. A tissue sample was taken for laboratory analysis and was sent for cultures and histopathology. Lateral mass screws were inserted from C3-6 bilaterally excluding C4 due to difficult entry. Homeostasis was maintained, bone graft applied over lateral mass and wound was closed over a drain. Intraoperative cultures did not grow any bacteria. The surgical pathology report revealed a histological feature consistent with an organizing hematoma. 

The patient’s initial post-operative recovery was slow, and neurological status remained the same until day five, then the patient started to improve. The patient was kept in our hospital for rehabilitation for two weeks, upon discharge motor examination of left shoulder abduction and elbow flexion was 2/5 and for wrist/hand power was 4/5, motor examination of left lower extremity was 4/5, right upper and lower extremity motor examination was 5/5. The patient’s latest follow-up three months after surgery showed full neurological recovery.

## Discussion

SSEH is an infrequent pathology in children, with only 29 cases having been reported. The majority of cases were in the cervical spine (17 out of 29; 58.6%) [3-5]. Clinically, the presentation of epidural hematoma in the pediatric age group varies significantly. Neck pain, abnormal neck position, focal or complete neurological weakness, numbness depending on level and location of blood accumulation, irritability or abnormal crying in infants has been reported [7,14]. The rate of symptom progression is typically fast, however slower progression of neurological symptoms has been seen. As a result of this ambiguous presentation, a correct diagnosis of this etiology is difficult.

Appropriate laboratory tests including platelet count and coagulation panel are essential to rule out any coagulation disorders or haemorrhagic diathesis. The cause of bleeding could not be identified. Although at the start of symptoms our patient was diagnosed and treated as a case of meningitis which was ruled out after final result of intra-operative culture and histology report, spontaneous spinal epidural hematoma mimicking meningitis was reported in previous cases [10]. The spinal MRI with contrast is considered the best available method to pick up such lesions [15,16]. The early MRI detection of hematoma offers a great chance for early treatment, leading to optimal neurological recovery. The hematoma typically appears isointense on T1-weighted and hyper-intense on T2-weighted MRI in the first 24 hours from onset of symptoms while it often appears hyper-intense on both T1- and T2-weighted images 24 hours later and may appear hypo-intense on both T1- and T2-weighted images in chronic hematoma [15]. In our case the hematoma appeared isointense on T1-weighted and hyper-intense on T2-weighted although it was performed after seven days of onset of symptoms. 

The mainstay of treatment is urgent surgical decompression and evacuation of accumulated blood from neural structures [3,17]. Although the main factor determining the prognosis was the pre-operative neurological status and not the onset of the symptoms, urgent surgical intervention and decompression should be considered in all symptomatic patients, especially those who present with incomplete cord injury given that these patients have higher chances of full recovery and resolution of neurological deficit [2].

A review of 29 pediatric cases reported by Pecha and Smeets showed complete resolution of neurological deficits in 15 out of 29 (51.7%) patients, partial resolution in 13 (44.8%) patients, and death in one (3.4%) patient [3,5]. In the present report, the child showed partial recovery after five days from laminectomy and evacuation of hematoma and full recovery was reached after three months.

## Conclusions

A high index of clinical suspicion followed by full investigation including an urgent MRI is crucial in diagnosing SSEH. Urgent surgical intervention is generally recommended as a first-line treatment followed by extensive rehabilitation program. This case was reported to be added to previously reported cases to give attention to the existence of this unique condition and avoid missing patients with such presentation.
